# Menthol, an Anti-Neuralgic

**Published:** 1880-01

**Authors:** A. D. Macdonald


MENTHOL, AN ANTI-NEURALGIC.—Allow me to mention that in neuralgia of the face, I have several times applied a solution of menthol, a solid derived from the Chinese or American oil of peppermint, referred to in *The Lancet* of 7th June last, as a powerful antiseptic and theoretically anti-neuralgic agent. The solution used was, on a first trial, one of the melted crystals only; but to avoid the annoyance to the eyes from the extreme volatility of the remedy, and to obtain a more prolonged action, I afterwards used a mixture of menthol in rectified spirit, with the addition of a little clove oil, to a strength of one of menthol to sixty parts, painted over the affected tract. Relief was had in from two to four minutes, and within one or two minutes, at most, after this the then existing attack was cured. This, I think, goes far to show that the Chinese custom of painting with oil of peppermint in neuralgic cases, owes its reputed efficacy to menthol as its active constituent. In cases of toothache, the pain has disappeared within a few seconds after the application of a single crystal on cotton-wool. From this it is easy to go to sciatica, and I would recommend a trial of the crystal of menthol, melted, in this affection, as well as in intercostal neuralgia, brachialgia, and nerve pains in general.—*A. D. Macdonald, in the London Lancet.*

